# Injectable alginate–iridium complex hydrogel for sonodynamic therapy of breast cancer

**DOI:** 10.1039/d6ra05109j

**Published:** 2026-08-24

**Authors:** Jie Liu, Xuefen Zhao, Huiying Li, Shiyi Chai, Qiufang Gong, Peng Sun, Zhiyong Li, Yunyang Zhao, Chao Liang

**Affiliations:** a Department of Clinical Laboratory, The Second Affiliated Hospital and Yuying Children's Hospital of Wenzhou Medical University Wenzhou Zhejiang 325000 China liangchao@wmu.edu.cn; b Cixi Biomedical Research Institute, Wenzhou Medical University Ningbo Zhejiang 315300 China yunyangz@wmu.edu.cn; c School of Basic Medical Sciences, Wenzhou Medical University Wenzhou Zhejiang 325000 China sunpeng@wmu.edu.cn lizhiyong@wmu.edu.cn

## Abstract

Breast cancer remains one of the leading causes of cancer-related mortality in women worldwide, and sonodynamic therapy (SDT) has emerged as a promising non-invasive antitumor therapeutic modality. In this work, an injectable alginate (ALG) hydrogel loaded with an iridium complex (Ir958) was rationally fabricated for SDT-mediated suppression of breast cancer growth. The optimized composite hydrogel exhibited favorable injectability and excellent biocompatibility, while the loaded Ir958 effectively produced cytotoxic singlet oxygen upon ultrasound irradiation. *In vitro* biological evaluations demonstrated that the combined treatment of ALG/Ir958 hydrogel with ultrasound remarkably suppressed the proliferation of 4T1 breast cancer cells, triggered intracellular reactive oxygen species (ROS) overproduction, and ultimately facilitated tumor cell apoptosis. *In vivo* 4T1 xenograft experiments verified robust tumor suppression without major organ injury. Hypoxia-inducible factor 1α (HIF-1α) immunofluorescence and hematoxylin and eosin (H&E)/TUNEL staining further confirmed the ROS-mediated antitumor mechanism and tumor cell apoptosis. Collectively, this hydrogel is a safe and effective SDT platform for breast cancer with great clinical translation potential.

## Abstract 2

An alginate hydrogel loaded with an iridium complex was rationally fabricated for SDT-mediated suppression of breast cancer growth.

## Graphical abstract

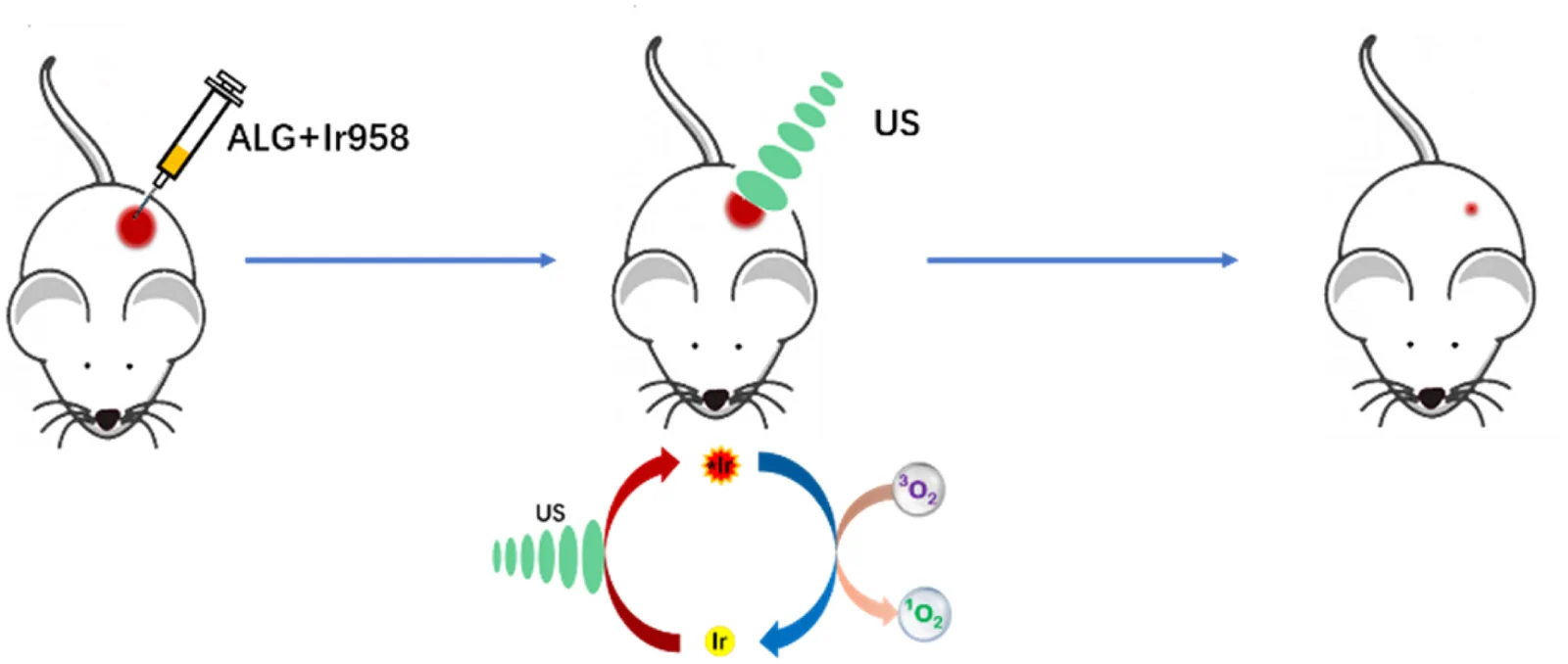

## Introduction

Cancer, also known as malignant tumors, is characterized by abnormal cell proliferation, invasion, and metastasis, posing a severe threat to human health.^1,2^ According to recent statistics, cancer has become the leading cause of death worldwide.^3^ Conventional antitumor therapies such as chemotherapy, radiotherapy, and surgical resection often suffer from limitations including poor targeting, severe side effects, drug resistance, and high recurrence rates, which significantly affect treatment outcomes and patient quality of life.^4^ Thus, there is an urgent need to develop novel non-invasive, safe, and targeted therapeutic strategies for cancer treatment.

Sonodynamic therapy (SDT), first proposed by Japanese scientist Yumita, is a promising non-invasive antitumor technology derived from photodynamic therapy (PDT).^5^ Similar to PDT, SDT relies on three essential components: ultrasound (US), oxygen, and sonosensitizers. Under ultrasonic irradiation, sonosensitizers are activated to generate reactive oxygen species (ROS), primarily singlet oxygen (^1^O_2_), which induce tumor cell apoptosis, necrosis, or autophagy.^6,7^ Compared with traditional therapies and PDT, SDT offers unique advantages including deep tissue penetration, precise targeting *via* adjustable ultrasound parameters, and minimal damage to normal tissues.^4,8^ SDT was reported for the treatment of advanced refractory breast cancer patients unresponsive to conventional therapies.^9,10^ However, the exact mechanism of SDT is not yet fully elucidated, and the development of efficient sonosensitizers remains a key challenge.

Platinum group metals (PGMs) have gained significant attention in cancer therapy due to their unique chemical properties and antitumor activities.^11^ Among PGMs, iridium complexes exhibit several superior characteristics compared to traditional platinum drugs, such as higher tumor selectivity, lower toxicity, enhanced stability under physiological conditions, and excellent photophysical properties.^12–15^ Moreover, Ir complexes can act as efficient sonosensitizers to generate ROS under ultrasonic stimulation, making them promising candidates for SDT.^16,17^

Alginate (ALG), a natural anionic polysaccharide extracted from brown algae, has been widely used as a drug delivery carrier due to its biocompatibility, biodegradability, low toxicity, and easy gelation.^18–20^ Injectable alginate hydrogels, formed *via* ionic crosslinking with divalent cations (*e.g.*, Ca^2+^), can be directly administered to tumor sites, providing sustained drug release and improved targeting. This localized delivery system can enhance the accumulation of sonosensitizers at the tumor site, thereby improving SDT efficacy and reducing systemic side effects.^21,22^ Despite the combined merits of iridium sonosensitizers and alginate carriers, most existing hydrogel-sonodynamic platforms simply physically encapsulate sonosensitizers within gel networks. After intratumoral injection, the loaded agents readily diffuse and leak out of the hydrogel matrix, resulting in insufficient intratumoral retention and elevated risks of off-target systemic toxicity. Differing markedly from these conventional physical blending strategies, iridium complex possesses favorable compatibility with alginate networks and can be stably immobilized inside the hydrogel scaffold to achieve long-term tumor-site retention.

In this study, we fabricated an injectable alginate hydrogel loaded with iridium complex (Ir958, denoted as “Ir” in group nomenclature) for SDT of breast cancer. The formulation achieves stable embedding of Ir958 within the alginate network to sustain long-term intratumoral retention and persistent *in situ* ROS production upon US stimulation. Comprehensive material characterizations covering microstructure morphology and injectability were performed, alongside systematic *in vitro* and *in vivo* assays to validate the hydrogel's biocompatibility, sonodynamic antitumor performance and systemic biosafety. Collectively, experimental results verified that the combined ALG/Ir hydrogel-SDT strategy remarkably suppresses primary breast tumor progression. This rationally optimized localized delivery platform addresses the retention deficiency of traditional hydrogel carriers and offers an innovative candidate therapeutic paradigm for breast cancer clinical intervention.

## Results and discussion

**Fig. 1:**
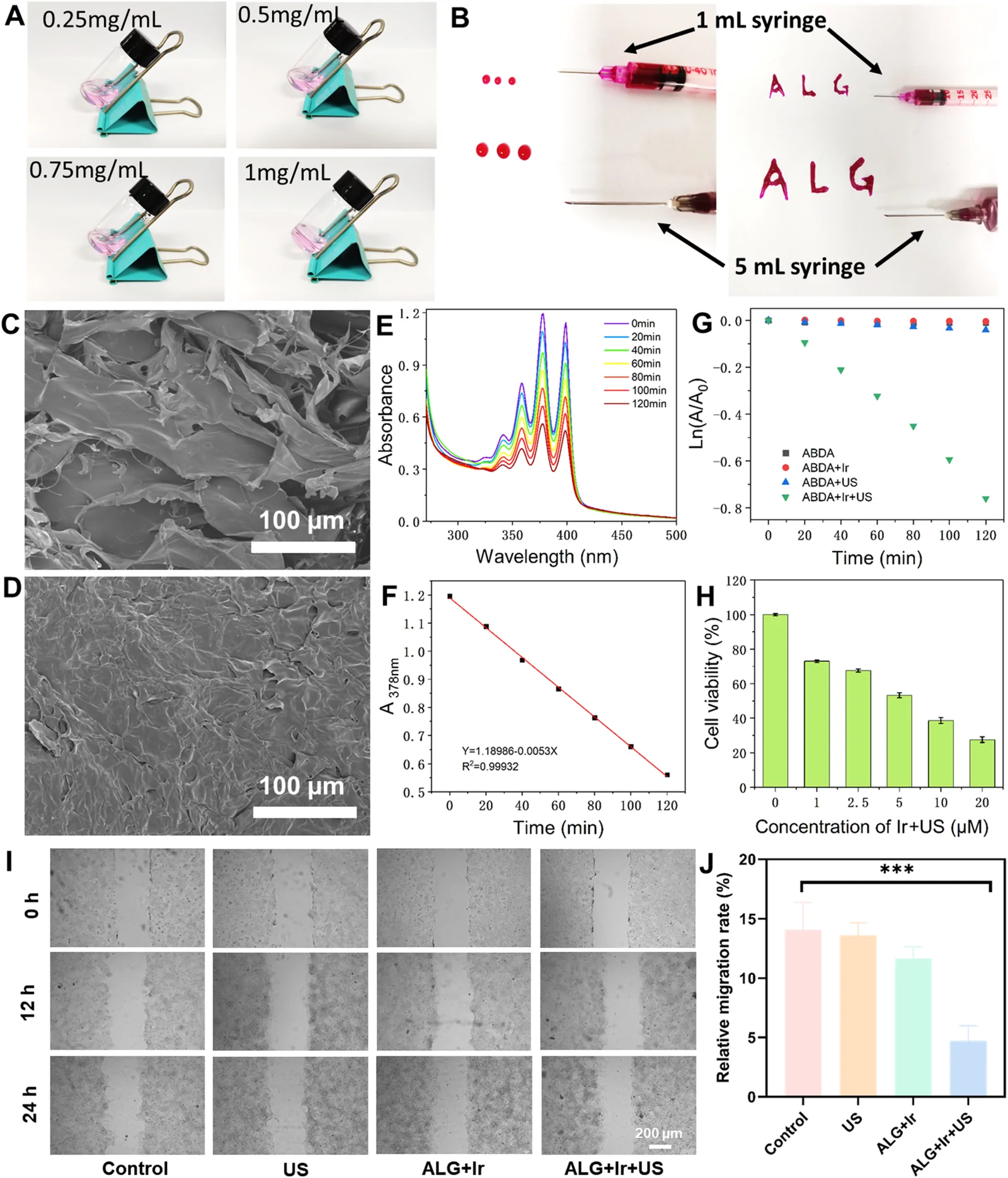
(A) The ALG hydrogel with different concentration of Ca^2+^, (B) images of the concentration-optimized injectable hydrogel (with 0.75 mg mL^−1^ Ca^2+^) extruded using 1 mL and 5 mL syringes, showing the droplet morphology and the formed “ALG” pattern. (C and D) The SEM images of ALG and ALG/Ir hydrogel, respectively. (E and F) The absorption spectra of ABDA in ABDA + Ir + US group with different times and the corresponding liner relationship between the absorbance at 378 nm with times. (G) Attenuation rate of ABDA at 378 nm under different experimental conditions. (H) Cell viability of 4T1 cells after 24 h co-incubation with Ir958 at different concentrations following US stimulation; (I) representative migration images of 4T1 cells from different treatment groups at the indicated time points, and (J) corresponding quantification of cell migration rate at 24 h (**P < 0.01, ***P < 0.001, *n* = 3). Migration rates were analyzed using ImageJ and plotted with GraphPad Prism 8.0.

The injectable ALG hydrogel was successfully fabricated *via* ionic crosslinking between alginate chains and Ca^2+^ ions. As shown in Fig. 1A, the results demonstrate the optimal Ca^2+^ concentration for favorable injectability is 0.75 mg mL^−1^. At Ca^2+^ concentrations below 0.75 mg mL^−1^, only a flowing liquid was formed, whereas higher concentrations led to non-injectable hydrogels. In Fig. 1B, the hydrogel formulated with the optimal CaCl_2_ concentration (0.75 mg mL^−1^) exhibited favorable injectability and could readily form the distinct pattern of “ALG” upon extrusion. The surface morphological features of the hydrogel were investigated *via* scanning electron microscopy (SEM). The representative SEM images in Fig. 1C and D showed that both the pristine ALG hydrogel and ALG with iridium complex (ALG/Ir) hydrogel had a porous structure, which is favorable for drug loading and release. While the addition of Ir958 altered the porous structure of the hydrogel to a certain degree, this structural modulation not only reflected good compatibility between Ir958 and the alginate matrix but also preserved an adequate specific surface area to support the performance requirements of subsequent SDT. We further quantified the loading efficiency of Ir958 within the ALG hydrogel and confirmed it reached nearly 100%. Corresponding *in vitro* release kinetic assays (Fig. S1, SI) demonstrated sustained, gradual cumulative release of Ir958 from the porous hydrogel over time.

The ROS generation capacity of Ir958 under ultrasonic stimulation was evaluated with 9,10-anthracenediyl-bis(methylene)dicarboxylic acid (ABDA) as probe. As illustrated in Fig. 1E–G and S2, the absorbance of ABDA at 378 nm decreased markedly with prolonged US stimulation time in the ABDA + Ir + US group, yielding a decomposition rate constant of 0.0053 min^−1^. In contrast, no obvious reduction in ABDA absorbance was observed in the other control groups. These results demonstrate that Ir958 can efficiently generate ^1^O_2_ upon ultrasonic activation, thereby validating its potential as an effective sonosensitizer for SDT.

The biocompatibility of the alginate hydrogel and Ir958 was evaluated using the 3-(4,5-dimethylthiazol-2-yl)-2,5-diphenyltetrazolium bromide (MTT) assay. As shown in Fig. S3A and B, the cell viability remained above 80% even at high concentrations of ALG hydrogel (30 mg mL^−1^) or Ir958 (50 µM), indicating their good biocompatibility and low toxicity. However, when combined with US activation, Ir958 significantly reduced cell viability in a concentration-dependent manner (Fig. 1H and S3C), with the highest cytotoxicity observed at 20 µM Ir958 (*P* < 0.001). These results suggested that the ALG/Ir hydrogel exhibited low cytotoxicity alone but could effectively kill tumor cells under ultrasonic stimulation, which is crucial for safe and effective SDT.^23^

Cell migration is a fundamental trait of tumor cells and a key indicator of anti-tumor efficacy. *In vitro* scratch assays were thus employed to evaluate 4T1 cell migration under various treatments as shown in Fig. 1I, J and S4. Under US stimulation, 4T1 cell migration gradually decreased with elevated Ir958 concentrations, and apparent cell death occurred at 20 µM Ir958 in Fig. S4A. Further investigation at 10 µM Ir958 revealed that the ALG + Ir + US group resulted in a much lower migration rate than other groups, confirming that this sonodynamic system efficiently inhibits 4T1 cell migration (Fig. 1I and J).

**Fig. 2:**
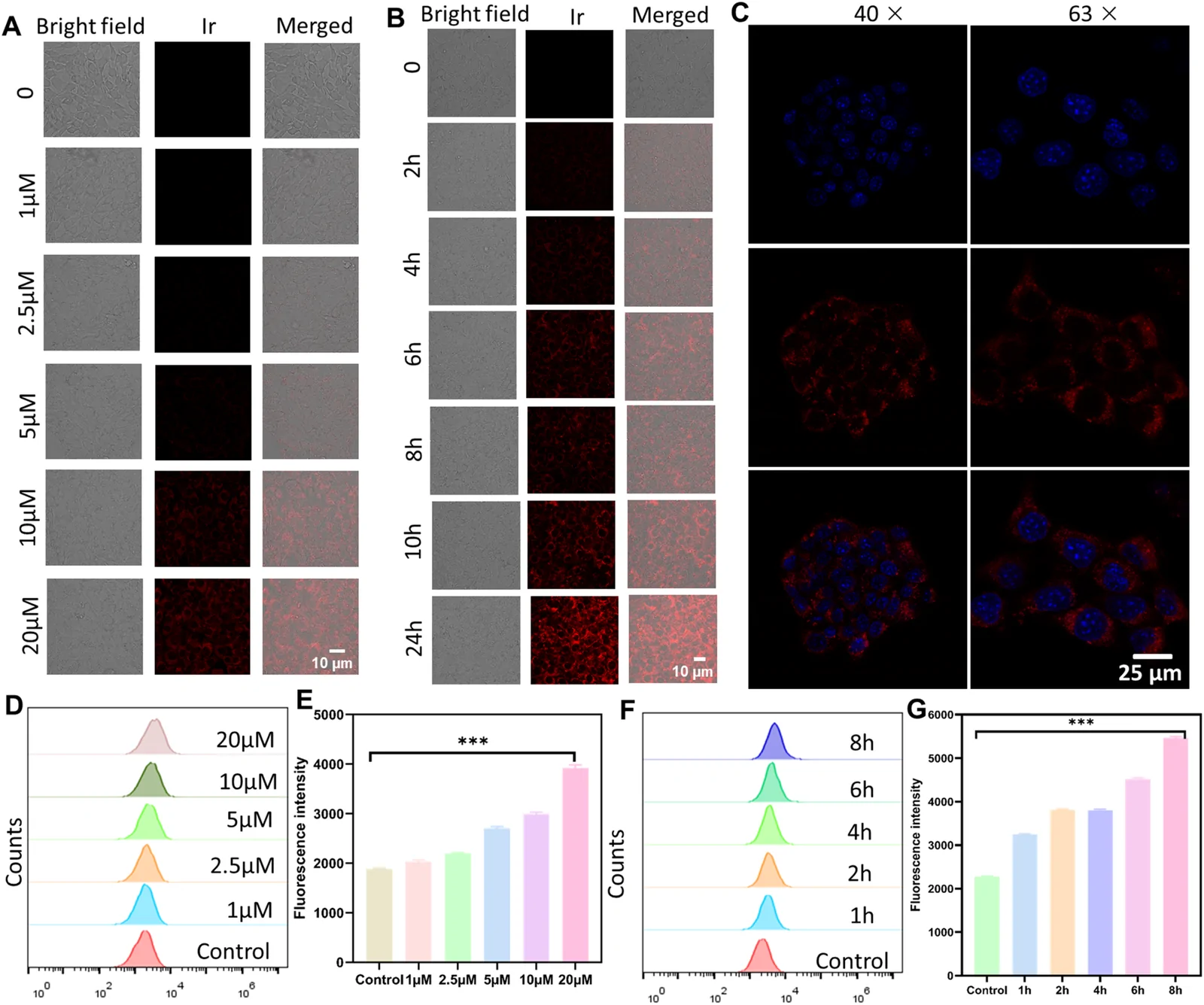
(A) Fluorescence images of 4T1 cells incubated with Ir958 at various concentrations for a fixed incubation time; (B) fluorescence images of 4T1 cells incubated with Ir958 at a constant concentration for different durations; (C) fluorescence images of 4T1 cells captured in multiple fields of view after incubation with 10 µM Ir958 for 4 h. Red fluorescence represents Ir958, and blue fluorescence indicates the nuclei stained by Hoechst 33258; (D) rightward-shifted fluorescence intensity curves for cells exposed to graded Ir958 concentrations at a fixed incubation time *vs.* control; (E) mean fluorescence intensity at various Ir958 concentrations with identical incubation duration; (F) rightward-shifted fluorescence intensity curves for cells treated with a fixed Ir958 concentration over increasing incubation time *vs.* control; (G) mean fluorescence intensity at diverse time points under a constant Ir958 dose (***P < 0.001, *n* = 3).

Cellular uptake assays of Ir958 were performed using confocal laser scanning microscopy (CLSM) and flow cytometry to determine the optimal incubation time and concentration for its cellular uptake. Given the intrinsic fluorescence of Ir958, CLSM visualize the fluorescence signal and evaluate the uptake profile in 4T1 cells as presented in Fig. 2A and B. The CLSM images revealed that the fluorescence intensity of Ir958 in 4T1 cells increased with drug concentration (Fig. 2A) and incubation time (Fig. 2B), indicating time- and concentration-dependent uptake. Based on the uptake and cytotoxicity data, 4 h incubation with 10 µM Ir958 was chosen for co-staining with Hoechst 33258. Bright, distinct fluorescence signals were detected in the cytoplasm across multiple fields of view, and colocalization analysis relative to Hoechst-stained nuclei validated efficient intracellular internalization and favorable fluorescence performance of Ir958 (Fig. 2C). Flow cytometry was employed to quantify the internalization of Ir958. As shown in Fig. 2D–G, the fluorescence distribution curves shifted rightward with prolonged incubation time and elevated Ir958 concentration, which further validated the observations from confocal imaging. These results confirm that the cellular uptake of Ir958 is time- and dose-dependent.

**Fig. 3:**
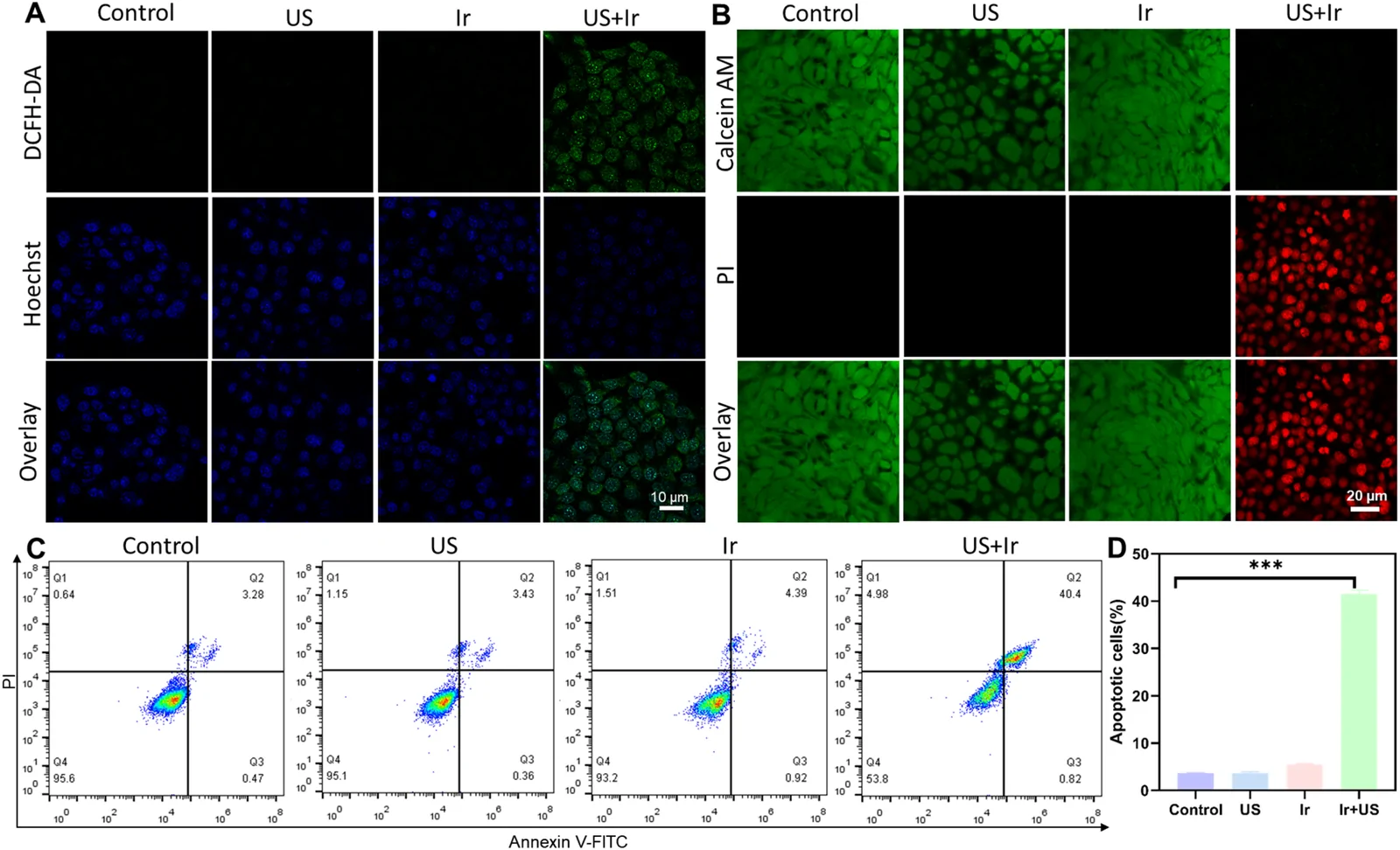
(A) CLSM images of DCFH-DA-stained 4T1 cells after different treatments (blue fluorescence: Hoechst 33258-stained cell nuclei); (B) CLSM images of four groups of 4T1 cells co-stained with Calcein AM and PI following various treatments; (C) apoptosis plots and (D) quantification analysis of 4T1 cells from different groups *via* Annexin V-FTC/PI staining for apoptosis analysis. (***P < 0.001, *n* = 3).

To validate intracellular ROS generation, we examined the ALG + Ir system under US stimulation in 4T1 cells using DCFH-DA (2′,7′-dichlorodihydrofluorescein diacetate, green fluorescence for ROS detection) as the ^1^O_2_ probe. As shown in Fig. 3A and S5, after co-incubation with Hoechst 33258 and DCFH-DA for 20 min, CLSM imaging revealed that the Ir + US group displayed markedly enhanced green fluorescence compared with the control, Ir, and US groups. This result confirmed that the synergistic effect of Ir958 and US induced robust ROS generation, which is the primary mechanism underlying SDT-mediated tumor cell killing.

Calcein AM/PI double staining assay was performed to evaluate the cytotoxic effect of Ir958 combined with US stimulation on 4T1 cells. In Fig. 3B, the cells were divided into four groups: control group, Ir group, US group, and Ir + US group. After respective treatments, the cells were co-stained with Calcein AM and propidium iodide (PI), followed by imaging under a CLSM. As shown in the imaging results, abundant green fluorescence (indicating viable cells) and no red fluorescence (indicating dead cells) were observed in the control group, Ir group, and US group, suggesting no significant cell death in these three groups. In contrast, the Ir + US group exhibited distinct red fluorescence with negligible green fluorescence. These results demonstrate that Ir958 combined with US stimulation induces massive 4T1 cell death, further confirming the significant cytotoxic effect of this SDT system on tumor cells.

To verify whether Ir958 could induce tumor cell apoptosis under US stimulation, an Annexin V/PI double staining assay was performed with flow cytometry for quantitative analysis of apoptotic cells. Flow cytometry analysis of apoptosis (Fig. 3C and D) revealed that the Ir + US group had a significantly higher apoptotic rate (40.4%) compared with the other groups (*P* < 0.001). These results collectively demonstrated that Ir + US induced tumor cell death primarily through apoptosis, mediated by ROS generation. Immunofluorescence staining of 4T1 cells was conducted using DAPI and caspase-3 probes across four experimental cohorts: control, US monotherapy, Ir monotherapy, and Ir + US combination groups. As showed in Fig. S6, robust green-fluorescent signals indicative of caspase-3 were uniquely observed in the combinatorial treatment group, whereas barely detectable signals appeared in the control and two single-treatment groups. This observation confirms that simultaneous administration of Ir958 and ultrasound is a prerequisite to activate caspase-3-mediated apoptotic pathways in tumor cells. Collectively, these data further verify that the antitumor potency of sonodynamic therapy stems from ultrasound-elicited ROS accumulation upon co-treatment with Ir958. The overproduced reactive oxygen species sequentially triggers caspase-3 upregulation and accelerates the apoptotic cascade of cancer cells. JC-1 mitochondrial membrane potential staining was performed to evaluate mitochondrial dysfunction during tumor cell apoptosis in four groups in Fig. S7. The control and single US groups exhibited predominantly red fluorescence, indicating intact mitochondrial membrane potential and normal cell physiological status. The Ir958 monotherapy group showed slightly increased green fluorescence, suggesting mild mitochondrial damage. In contrast, the combined treatment group displayed pervasive green fluorescence with negligible red signals, demonstrating severe mitochondrial depolarization. These results indicate that the synergistic SDT effect of Ir958 plus ultrasound markedly disrupts mitochondrial integrity, effectively inducing mitochondrial dysfunction and further triggering tumor cell apoptosis.

**Fig. 4:**
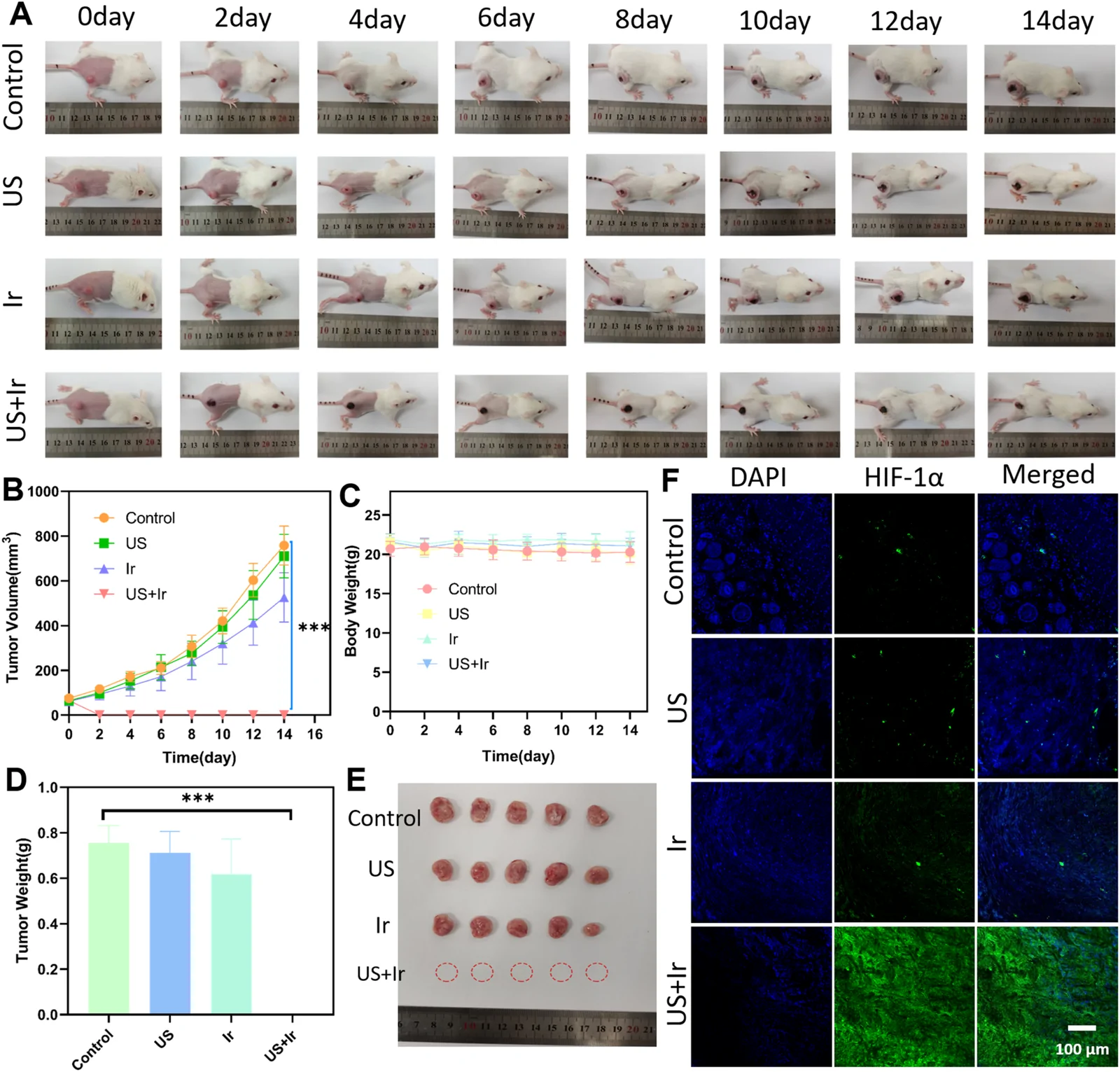
(A) Representative dorsal tumor images of BALB/c mice in different treatment groups captured every two days during treatment; (B) tumor volume growth curves of BALB/c mice in different groups recorded every two days during treatment; (C) body weight changes of BALB/c mice in different treatment groups; (D) tumor weights of excised tumors from different groups (***P < 0.001, *n* = 5); (E) representative images of dissected dorsal tumors from mice in different groups after 14 days of treatment; (F) comparison of intratumoral HIF-1α fluorescence intensity for hypoxia assessment in different groups.

To further verify the *in vivo* tumor inhibitory effect of the ALG/Ir hydrogel combined with SDT, animal experiments were conducted on BALB/c mice bearing subcutaneous 4T1 syngeneic tumors. The mice were randomly divided into four groups: control group, US group, Ir group, and Ir + US group. One week after tumor inoculation, when subcutaneous tumors reached an appropriate size, intratumoral injections were administered according to grouping, followed by US treatment. The body weight and tumor volume of mice in each group were measured every two days. 14 days after these different treatments, the mice were sacrificed, and subcutaneous tumors were excised and weighed. As shown in Fig. 4A–E, no significant difference in body weight was observed among the four groups, and all mice maintained a good physiological state, indicating that the treatment regimen caused no obvious systemic toxicity in mice.^24^ Notably, the Ir + US group exerted markedly superior tumor growth suppression relative to the other three groups (*P* < 0.001), while mild tumor volume shrinkage was also detected in single-treatment groups. This minor inhibitory effect can be ascribed to two factors: low-intensity ultrasound alone generates trace ROS *via* mechanical cavitation to slightly damage tumor cells, and individual differences in intrinsic antitumor immunity among BALB/c mice enable spontaneous mild suppression of 4T1 tumor proliferation in some individuals. As presented in Fig. S8, ALG hydrogel exerted a favorable immobilization effect, which prolonged the retention of Ir958 at the tumor site, enhancing the SDT efficacy.

For hypoxia detection, mice were sacrificed at 24 h post intratumoral injection, and tumor tissues were collected for frozen sectioning and immunofluorescence staining with hypoxia-inducible factor-1α (HIF-1α) antibody, a canonical marker of hypoxia induced by ROS. As shown in Fig. 4F and S9, the Ir + US group displayed a markedly higher HIF-1α fluorescence signal than the other groups, indicating significant intratumoral hypoxia. This finding further confirms that the Ir + US system generates abundant ROS during US treatment, which is responsible for the potent tumor growth inhibitory effect. To further verify intratumoral ROS generation *in vivo*, tumor tissues harvested from four groups of 4T1-tumor-bearing mice were subjected to DCFH-DA immunofluorescence staining (Fig. S10). Consistent with the *in vitro* findings, negligible green fluorescence signals were detected in the control, US monotherapy, and Ir958 monotherapy groups. In sharp contrast, robust green fluorescence was extensively observed in tumor tissues of the Ir + US combination group, indicating massive intratumoral ROS accumulation triggered by SDT treatment. These tissue-level results solidly confirm that the synergistic effect of Ir958 and ultrasound is essential for effective ROS production in tumor lesions, which acts as the core initiator of subsequent tumor apoptosis.

**Fig. 5:**
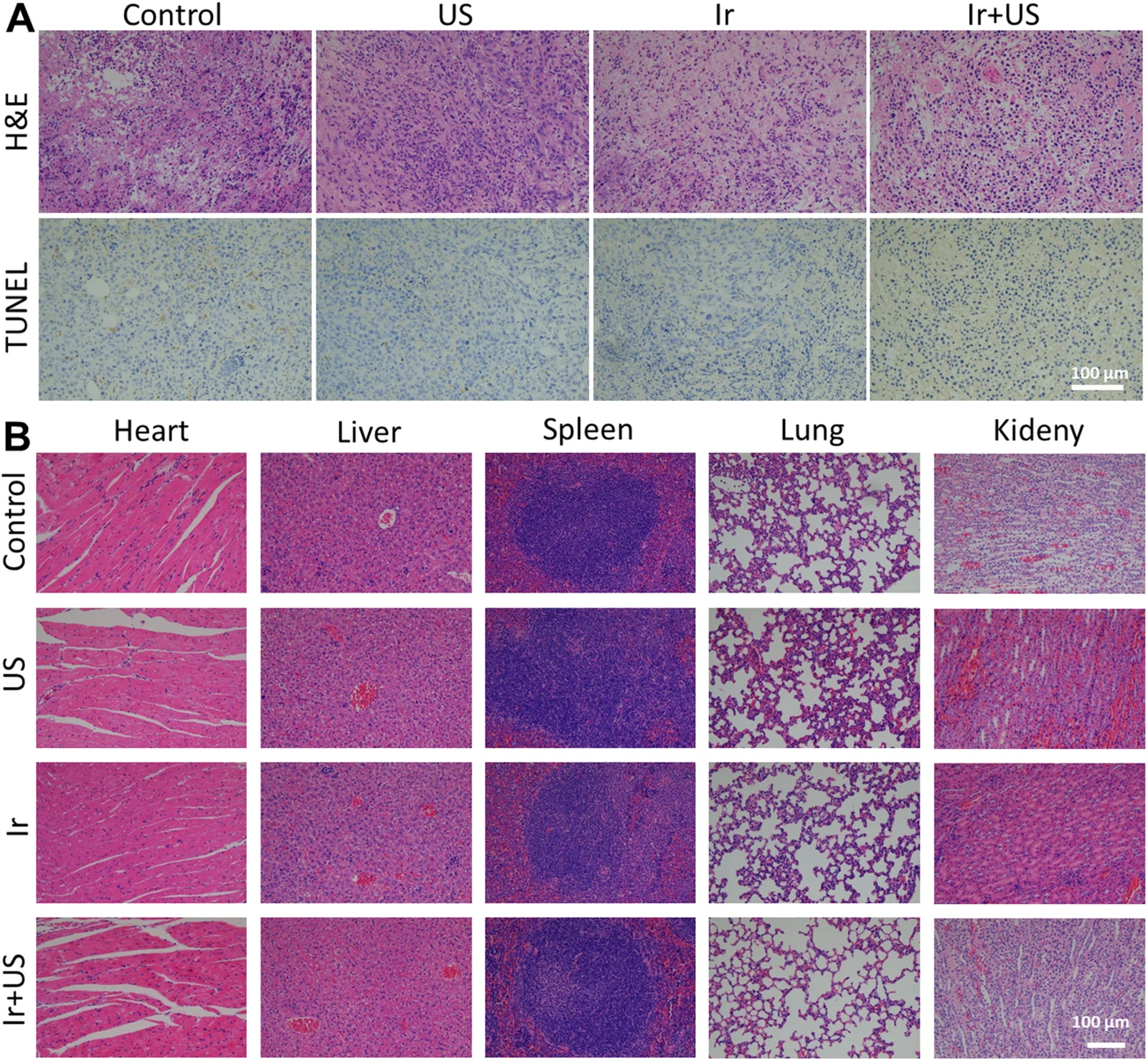
(A) H&E and TUNEL staining images of tumor tissues from BALB/c mice in different groups at 24 h post-treatment; (B) H&E staining images of major organs from BALB/c mice in different groups after 14 days of treatment.

At 24 h post-treatment, one mouse from each group was sacrificed, and tumor tissues were harvested for histological analysis. Tumor sections were stained with hematoxylin and eosin (H&E) and observed under a fluorescence microscope. To evaluate apoptosis induced by the treatment, tumor sections were further subjected to TUNEL staining and imaged under the same microscope. As shown in Fig. 5A, H&E staining revealed severe necrosis, nuclear shrinkage, and vacuolization in the tumor tissue of the Ir + US group, which were significantly more pronounced than in the other groups. Meanwhile, TUNEL staining showed remarkably more apoptotic cells in the US combined treatment group compared with other groups, consistent with *in vitro* apoptosis results and confirming the strong tumor-suppressive effect of Ir958 with US stimulation. To assess the *in vivo* biosafety, major organs (heart, liver, spleen, lung, and kidney) were collected from each group and stained with H&E. As displayed in Fig. 5B and S11, no obvious histological abnormalities or severe tissue damage were observed in any group, confirming the safety of the ALG/Ir958 hydrogel and SDT *in vivo*.

## Conclusions

In this study, an injectable ALG hydrogel loaded with Ir958 was fabricated for the SDT of breast cancer. The optimized hydrogel showed favorable injectability, biocompatibility and porous structure, and Ir958 could effectively generate ^1^O_2_ under US. *In vitro*, ALG/Ir958 + US notably suppressed 4T1 cell proliferation, induced ROS overproduction and apoptosis. *In vivo*, it markedly inhibited tumor growth without major organ damage, with enhanced tumor localization of Ir958. Collectively, this injectable hydrogel represents a safe and efficient sonodynamic therapeutic platform for breast cancer, exerting antitumor effects with promising prospects for clinical translation.

## Conflicts of interest

The authors declare no competing interests.

## Supplementary Material

RA-OLF-D6RA05109J-s001

## Data Availability

The all data generated in this study have been deposited in the Zenodo. Other data related to this work are available from the corresponding authors upon reasonable request. Supplementary information (SI): the experimental section and Fig. S1–S11. See DOI: https://doi.org/10.1039/d6ra05109j.
