# Supplementary material for: Injectable alginate–iridium complex hydrogel for sonodynamic therapy of breast cancer
Source: RSC Adv. 2026 Aug 24;16(43):52471–8. doi: 10.1039/d6ra05109j (PMC13502236; doi:10.1039/d6ra05109j)
Supplement: Supplementary file 1 [file RA-016-D6RA05109J-s001.pdf]

Supplementary Information (SI) for RSC Advances.
This journal is © The Royal Society of Chemistry 2026

Supporting information

2.1 Materials

BALB/c mice (18-20 g) were purchased from the Experimental Animal Center of Wenzhou Medical University (Wenzhou, China) and housed in a specific pathogen-free (SPF) environment. The animal experiments were approved by the Ethics Committee of Wenzhou Medical University (approval number: SYXK (Zhe) 2022-0034). 4T1 breast cancer cells were obtained from the Cell Bank of Chinese Academy of Sciences (Shanghai, China) and cultured in RPMI 1640 medium supplemented with 10% fetal bovine serum (FBS) and 1% penicillin-streptomycin at 37°C in a 5% CO_2 incubator.

Iridium complex (Ir958) was synthesized as previously reported [32]. Alginate (low viscosity) was purchased from J&K Scientific (Beijing, China). Calcium chloride ($CaCl_2$), ABDA (9,10-anthracenediyl-bis(methylene)dicarboxylic acid), and other chemical reagents were obtained from Sigma-Aldrich (St. Louis, USA) or equivalent suppliers. Cell culture reagents, including RPMI 1640 medium, FBS, and trypsin-EDTA, were purchased from Gibco (Grand Island, USA). ROS probe (DCFH-DA), apoptotic detection kit (Annexin V-FITC/PI), and live/dead staining kit (Calcein AM/PI) were obtained from Beyotime Biotechnology (Shanghai, China). HIF-1α antibody and Alexa Fluor® 488-conjugated secondary antibody were purchased from Abcam (Cambridge, UK) and Jackson Immuno Research (West Grove, USA), respectively.

Major instruments used included an ultrasonic therapeutic apparatus (EMS Physio Ltd, UK), UV-visible (UV-vis) spectrophotometer (Youke, China), scanning electron microscope (SEM, Hitachi, Japan), confocal laser scanning microscope (Leica, Germany), flow cytometer (BD Biosciences, USA), and enzyme-linked immunosorbent assay (ELISA) reader (Molecular Devices, USA).

2.2 Preparation and Characterization of ALG/Ir958 Hydrogel

2.2.1 Hydrogel Formation

Alginate solutions (20 mg/mL) were prepared by dissolving alginate powder in deionized water, and the pH was adjusted to 7.4 using HCl or NaOH. $CaCl_2$ solutions with different concentrations (0, 0.5, 1.0, 1.5, 2.0 mg/mL) were mixed with rhodamine B (1 mg/mL) for visualization. Equal volumes of alginate solution and $CaCl_2$ solution were mixed under gentle stirring, and the gelation behavior was observed. The optimal concentrations of ALG and $CaCl_2$ for injectable hydrogel formation were determined.

2.2.2 Preparation of ALG/Ir958 Hydrogel

Based on the optimal concentrations, Ir958 was added to the alginate solution under dark conditions to obtain a final Ir958 concentration of 10 μM. Equal volumes of the alginate-Ir958 mixture and $CaCl_2$ solution (0.75 mg/mL) were mixed to form the ALG/Ir958 hydrogel.

2.2.3 SEM Characterization

The hydrogels were frozen at -80°C for 24 h and then lyophilized using a freeze dryer. The dried hydrogels were cut into thin slices, sputter-coated with gold, and observed under a SEM at an accelerating voltage of 10 kV.

2.2.4 ROS Generation Assay

The ROS generation capacity of Ir958 under ultrasonic irradiation was evaluated using ABDA as a $^1O_2$ probe. Ir958 (10 μM) was mixed with ABDA (100 μM) in PBS. The mixture was irradiated with pulsed ultrasound at 3 MHz with a peak intensity of 3 W/cm^2 (10% duty cycle) for 20 min, and UV-visible absorption spectra (200-700 nm) were recorded every 20 min for 120 min. Control groups included ABDA alone, ABDA + ultrasound (US), and ABDA + Ir958 without US. The decomposition rate of ABDA at 378 nm was calculated to evaluate $^1O_2$ generation.

2.2.5 Determination of Ir958 Loading Efficiency

The loading efficiency of Ir958 in ALG/Ir958 hydrogel was determined via UV-vis spectrophotometry. Briefly, the Ir958 stock solution was uniformly mixed with sodium alginate aqueous solution, and calcium chloride solution was subsequently added to crosslink the mixture and form homogeneous ALG/Ir958 composite hydrogel. After complete gelation, the resultant hydrogel mixture was centrifuged at 2000 rpm for 10 min to separate and collect unencapsulated free Ir958 solution. The supernatant was subjected to UV-vis detection at the characteristic absorption wavelength of Ir958. However, No free Ir958 solution (no supernatant) was collected after centrifugation, indicating that almost all Ir958 was successfully encapsulated within the AGL hydrogel network, corresponding to an approximately 100% loading efficiency.

2.2.6 In Vitro Drug Release Rate Profile of ALG/Ir958 Hydrogel

The in vitro release profile of Ir958 from ALG/Ir958 hydrogel was measured by static immersion method. Briefly, the prepared ALG/Ir958 hydrogel was immersed in an equal volume of normal saline and incubated under stationary conditions. A 100 μL aliquot of the release medium was collected at hourly intervals throughout the experiment. The absorbance values of the collected release media was measured using a UV-Vis spectrophotometer at the visible characteristic absorption band of Ir958. The drug release rate was calculated with the equation as follows:

$$Release\ rate = \frac{A_x - A_0}{A_1 - A_0} * 100\%$$

where A_x, A_1, and A_0 represent the absorbance of the withdrawn solution at a given time point, the absorbance corresponding to the concentration of complete Ir958 release, and the absorbance of pure saline, respectively.

Finally, the in vitro release rate profile of Ir958 from the hydrogel was plotted with release time as the abscissa and cumulative release rate as the ordinate.

2.3 In Vitro Cell Experiments

2.3.1 Cell Culture

4T1 cells were cultured in RPMI 1640 complete medium at 37°C with 5% CO_2. Cells were passaged every 2-3 days when reaching 80% confluence. For experiments, cells were seeded in 96-well plates (8×10^3 cells/well), 6-well plates (2×10^5 cells/well), or confocal dishes (2.5×10^5 cells/dish) and cultured for 24 h before treatment.

2.3.2 Cytotoxicity Assay

Cells were treated with different concentrations of alginate hydrogel (0-30 mg/mL) or Ir958 (0-50 μM) for 24 h. For SDT groups, cells were treated with Ir958 (0-20 μM) followed by pulsed ultrasonic irradiation (3 MHz, 3 W/cm^2 with 10% duty cycle, 20 min) and further cultured for 24 h. Cell viability was measured using the MTT assay. The absorbance was recorded at 490 nm using an ELISA reader, and cell viability was calculated as $(A - A_0)/(A_c - A_0) \times 100\%$, where A, Ac, and A_0 represent the absorbance of the experimental, control, and blank groups, respectively.

PS: Unless otherwise specified, all in vitro cellular experiments in this study were performed using free Ir958 without the alginate hydrogel carrier, to avoid potential interference of the hydrogel matrix on cellular behavioral and functional characterization.

2.3.3 Cell Migration Assay (Wound Healing Assay)

Cells were seeded in 6-well plates and cultured until confluent. A sterile 10 μL pipette tip was used to create a straight wound. After washing with PBS to remove cell debris, cells were treated with Ir958 (0-20 μM) followed by US irradiation, or divided into four groups: control, Ir958 alone, US alone, and Ir958 + US. Wound images were captured at 0, 12, and 24 h using an inverted microscope. The wound closure rate was calculated using ImageJ software.

2.3.4 Cellular Uptake Assay

2.3.4.1 Confocal Microscopy

Cells were seeded in confocal dishes and treated with Ir958 (10 μM) for different time points (0, 2, 4, 6, 8, 10, 24 h) or different Ir958 concentrations (0-30 μM) for 4 h.

Nuclei were stained with Hoechst 33258 (5 μg/mL) for 15 min before imaging. Fluorescence images were captured using a confocal microscope with a 63× oil immersion objective.

2.3.4.2 Flow Cytometry

Cells were treated with Ir958 (10 μM) for different time points (0-10 h) or different concentrations (0-20 μM) for 4 h. After washing with PBS, cells were harvested and analyzed using a flow cytometer. The mean fluorescence intensity (MFI) was recorded to evaluate cellular uptake.

2.3.5 Intracellular ROS Detection

Cells were divided into four groups: control, Ir958 alone, US alone, and Ir958 + US. After treatment, cells were incubated with DCFH-DA (10 μM) for 20 min. Nuclei were stained with Hoechst 33258, and fluorescence images were captured using a confocal microscope.

2.3.6 Live/Dead Staining Assay

Cells were treated as described above and stained with Calcein AM (2 μM) and PI (5 μM) for 20 min. Live cells (green fluorescence) and dead cells (red fluorescence) were observed under a confocal microscope.

2.3.7 Apoptosis Assay

Cells were treated and harvested, then stained with Annexin V-FITC and PI according to the kit instructions. Apoptotic cells were analyzed using a flow cytometer. The percentage of apoptotic cells (early + late apoptosis) was calculated.

2.4 *In Vivo* Animal Experiments

2.4.1 Tumor Model Establishment

BALB/c mice were subcutaneously injected with 4T1 cells (5×10^6 cells/50 μL) into the dorsal region. When tumors reached approximately 5×5 mm in size (7 days after inoculation), mice were randomly divided into four groups (n=5 per group): control, ALG/Ir958 alone, US alone, and ALG/Ir958 + US.

2.4.2 Hydrogel Localization Assay

Two tumor-bearing mice received intratumoral injections of free Ir958 mixed with 1,1'-dioctadecyl-3,3,3',3'-tetramethylindodicarbocyanine (DiD) or ALG/Ir958 co-loaded with DiD. The visible blue staining within tumor tissue was tracked macroscopically to assess the intratumoral retention and localization of the alginate hydrogel carrier.

2.4.3 *In Vivo* ROS Detection

Mice were treated as grouped, and 24 h later, tumors were excised, embedded in OCT, and frozen at -80°C. Frozen sections (5 μm) were fixed with acetone, blocked with 5% skim milk, and incubated with HIF-1α primary antibody (1:100) overnight at 4°C. After washing, sections were incubated with Alexa Fluor® 488-conjugated secondary antibody (1:500) for 1 h, stained with DAPI, and observed under a confocal microscope.

Tumor tissues were isolated from mice after different treatments, rinsed with pre-cooled phosphate-buffered saline (PBS) to remove surface blood and impurities, and then embedded in OCT compound. The embedded tumor samples were frozen at −20 °C and cut into continuous frozen sections with a thickness of 8–10 μm using a freezing microtome. The obtained frozen sections were thawed at room temperature and washed with PBS to remove residual OCT embedding medium.

For intracellular ROS detection, the tumor sections were incubated with 10 μM DCFH-DA working solution in the dark for 20 min at 37 °C, consistent with the in vitro cell staining conditions. After incubation, the sections were rinsed three times with PBS to eliminate unbound fluorescent probes. Subsequently, cell nuclei were counterstained with DAPI staining solution for 5 min in the dark. After thorough washing with PBS to remove redundant staining solution, the sections were sealed with anti-fade mounting medium.

The fluorescence distribution of ROS in tumor tissues was observed and photographed using a laser scanning confocal microscope. The green fluorescence

intensity of DCFH-DA reflected the in situ ROS generation level in tumor tissues from different treatment groups.

2.4.4 Antitumor Efficacy Evaluation

Tumor volume and mouse body weight were measured every 2 days. Tumor volume was calculated as $V = L \times W^2/2$, where L and W are the longest and shortest diameters of the tumor, respectively. After 14 days of treatment, mice were euthanized, and tumors were excised, weighed, and photographed.

2.4.5 Histological Analysis

Tumor tissues and major organs (heart, liver, spleen, lung, kidney) were excised, fixed in 4% paraformaldehyde, embedded in paraffin, and sectioned (5 μm). Sections were stained with H&E for histological observation. TUNEL staining was performed on tumor sections to detect apoptotic cells according to the kit instructions. Stained sections were observed under an optical microscope.

2.5 Statistical Analysis

All data are presented as mean ± standard deviation (SD). Statistical analysis was performed using GraphPad Prism 8.0 software. One-way analysis of variance (ANOVA) followed by Tukey's post-hoc test was used to compare differences between groups. $P < 0.05$ was considered statistically significant.

Live subject statement

The study protocol involving animals was approved by the Institutional Animal Care and Use Committee (IACUC) of Wenzhou Medical University, and all experiments were performed in accordance with their ethical guidelines.

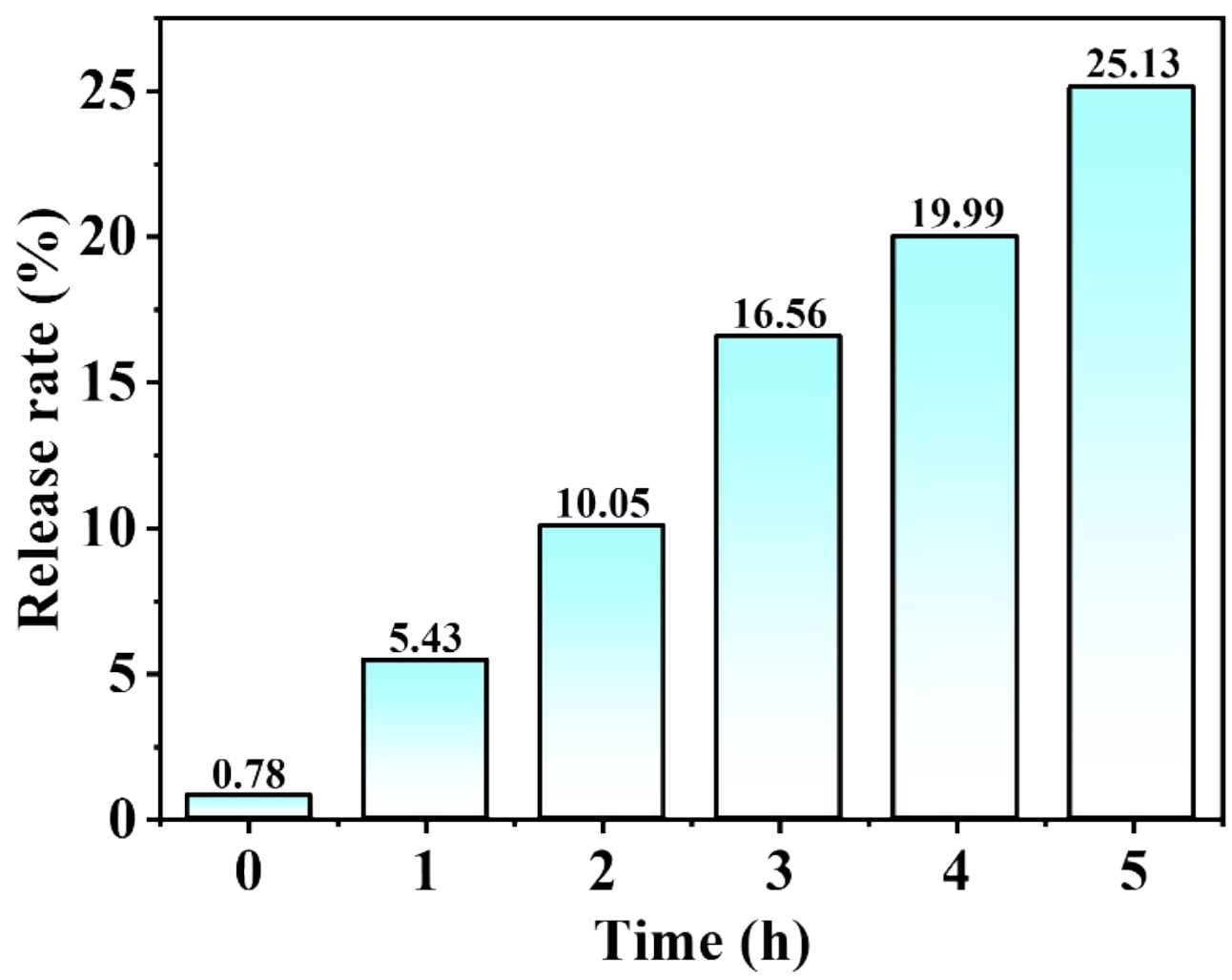

Figure S1. Time-dependent release profile of Ir958 from ALG hydrogel with near 100% loading efficiency.

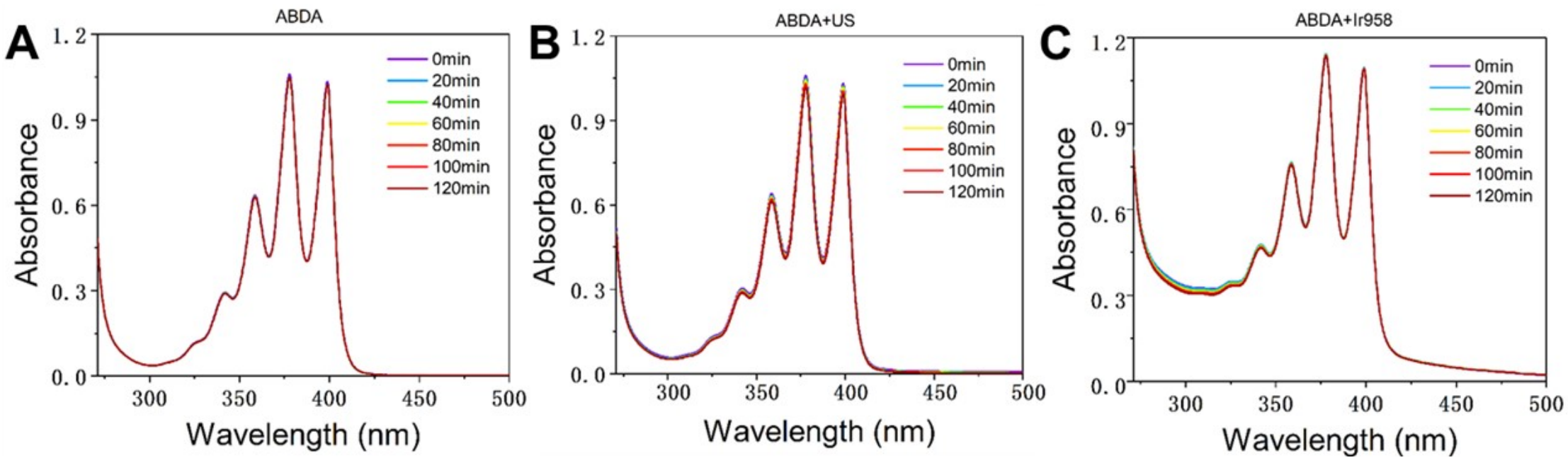

Figure S2. The absorption spectra of (A) ABDA without US stimulation; (B) ABDA with ultrasonic stimulation; (C) ABDA mixed with Ir958 in the absence of ultrasonic stimulation.

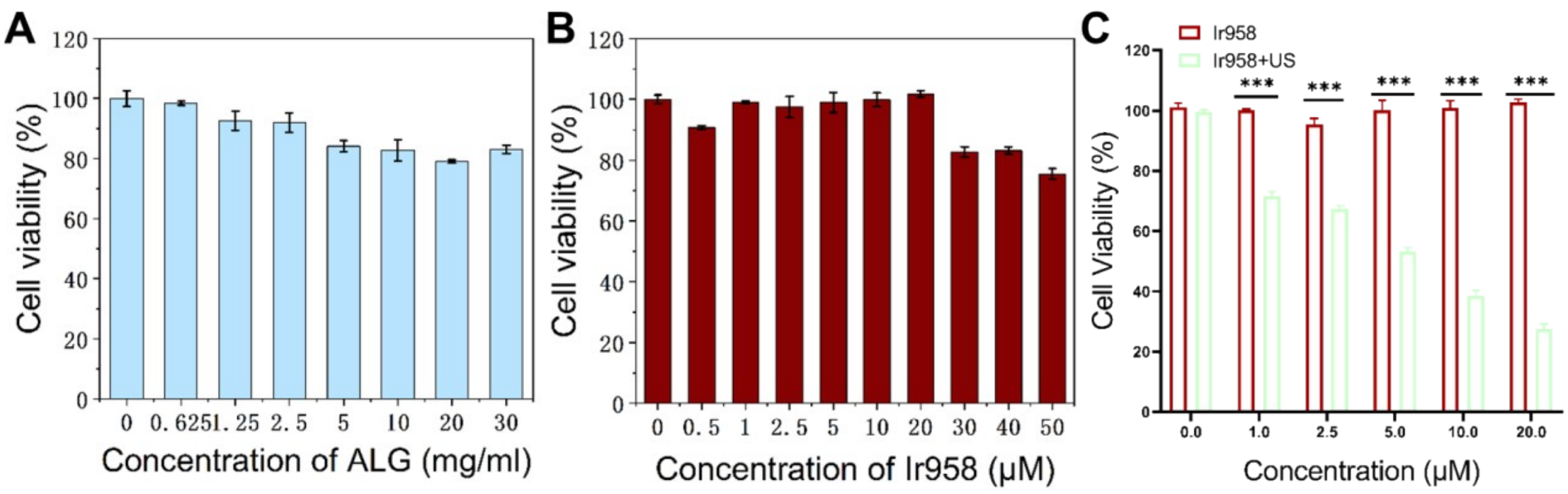

Figure S3 (A) Cell viability of 4T1 cells after 24 h co-incubation with ALG hydrogels at different concentrations; (B) Cell viability of 4T1 cells after 24 h co-incubation with Ir958 at various concentrations; (C) Cell viability of 4T1 cells following 24 h co-incubation with Ir958 at various concentrations, in the presence or absence of ultrasonic stimulation; (***$P < 0.001$, $n = 3$).

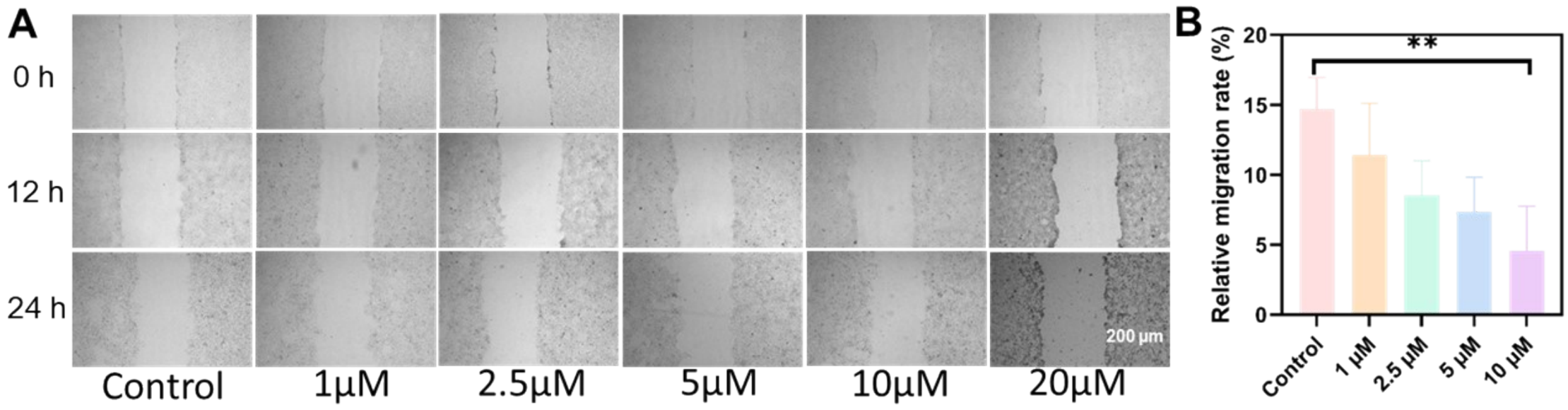

Figure S4. (A) Representative migration images of 4T1 cells treated with various concentrations of Ir958 under US at the indicated time points, and (B) corresponding quantification of 4T1 cell migration rate at 24 h. Migration rates were quantified using ImageJ and plotted with GraphPad Prism 8.0. (scale bar:200 nm)

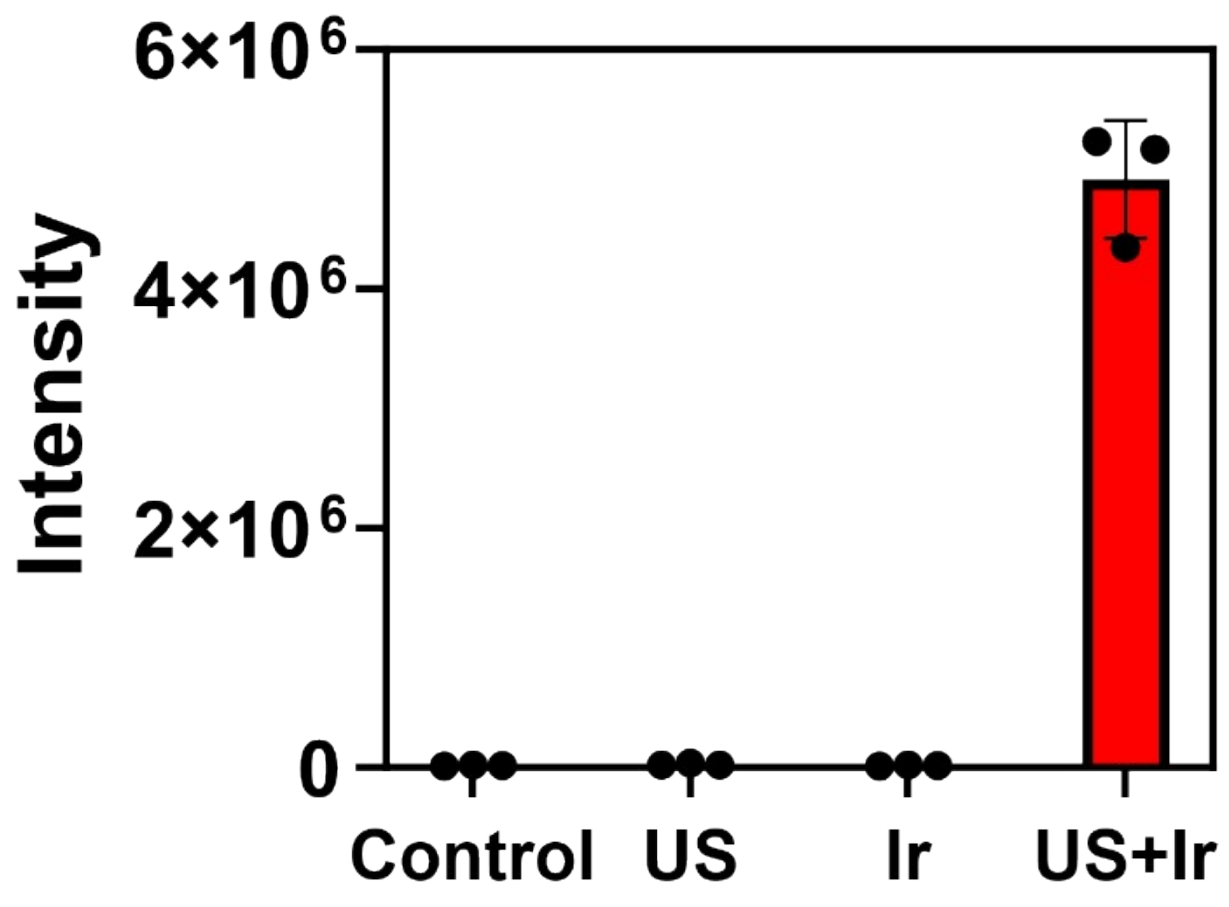

Figure S5. Comparison of green fluorescence intensity reflecting intracellular ROS levels in different groups.

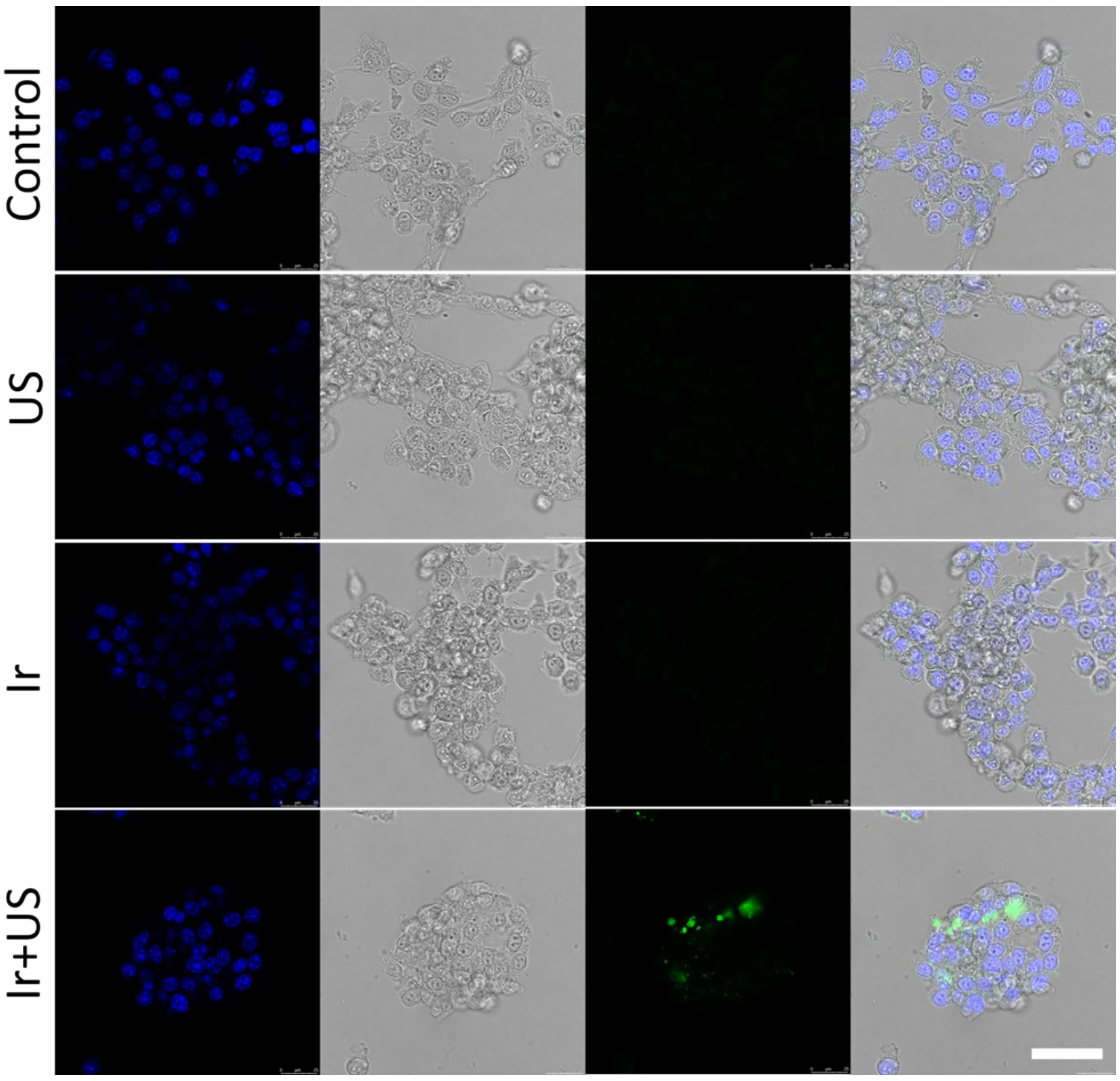

Figure S6. Immunofluorescence staining of caspase-3 in 4T1 cells under different treatments. Nuclei were stained with DAPI (blue), and activated caspase-3 was labeled with green-fluorescent probes. Groups: Control, US alone, Ir958 alone, Ir958 + US. Strong caspase-3 positive signals were only detected in the combined Ir958 and ultrasound group, verifying that sonodynamic therapy activates caspase-3-mediated tumor cell apoptosis. Scale bar: 50 μm

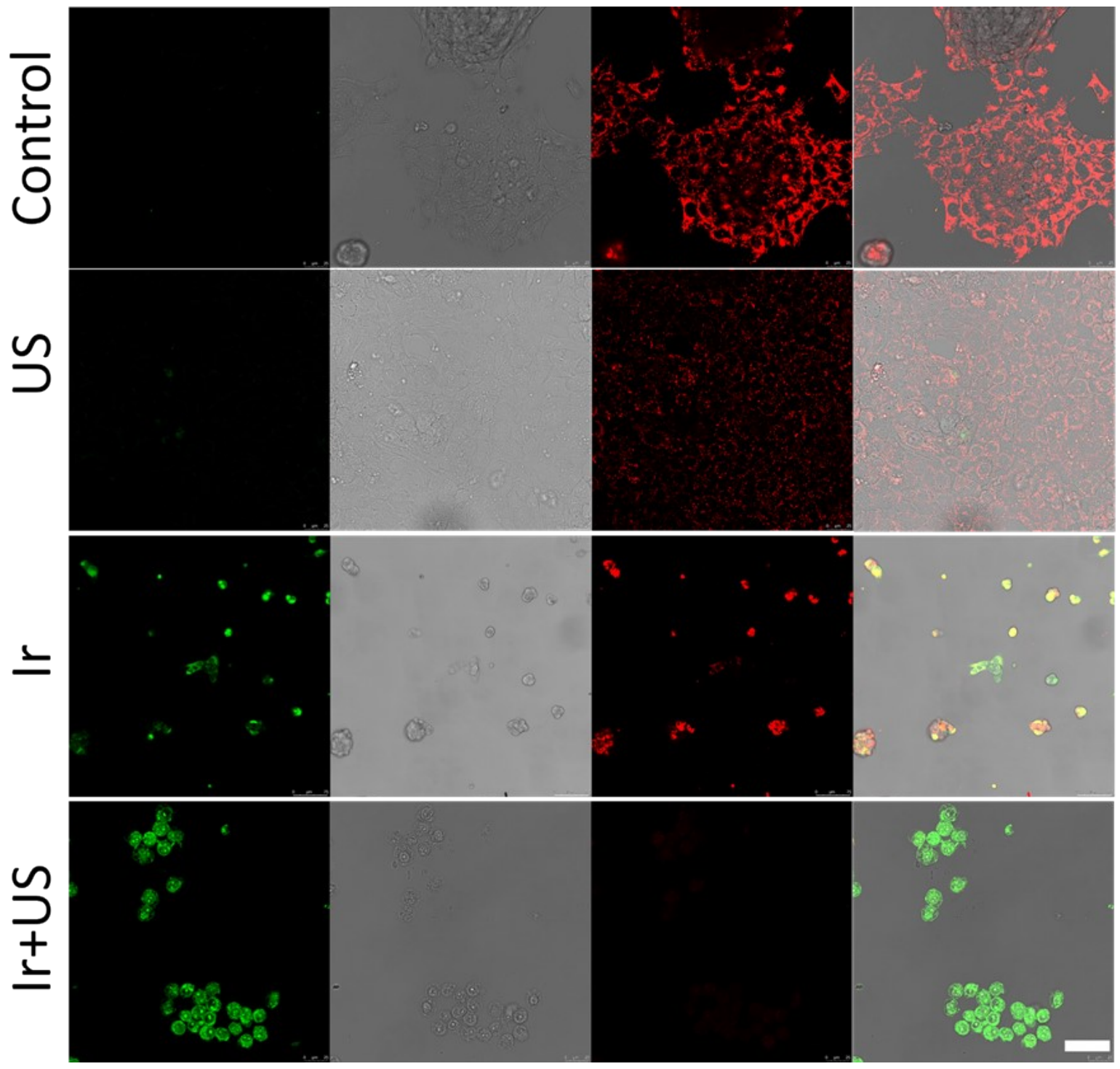

Figure S7. JC-1 staining for mitochondrial membrane potential of 4T1 cells after various treatments. Red fluorescence represents intact mitochondrial membrane potential, while green fluorescence indicates mitochondrial depolarization and damage. Control and US-only groups exhibited dominant red fluorescence; slight green signals appeared in the Ir958 single-treatment group, and widespread green fluorescence was observed in the Ir958 + US group, demonstrating severe mitochondrial dysfunction induced by synergistic sonodynamic therapy. Scale bar: 50 μm.

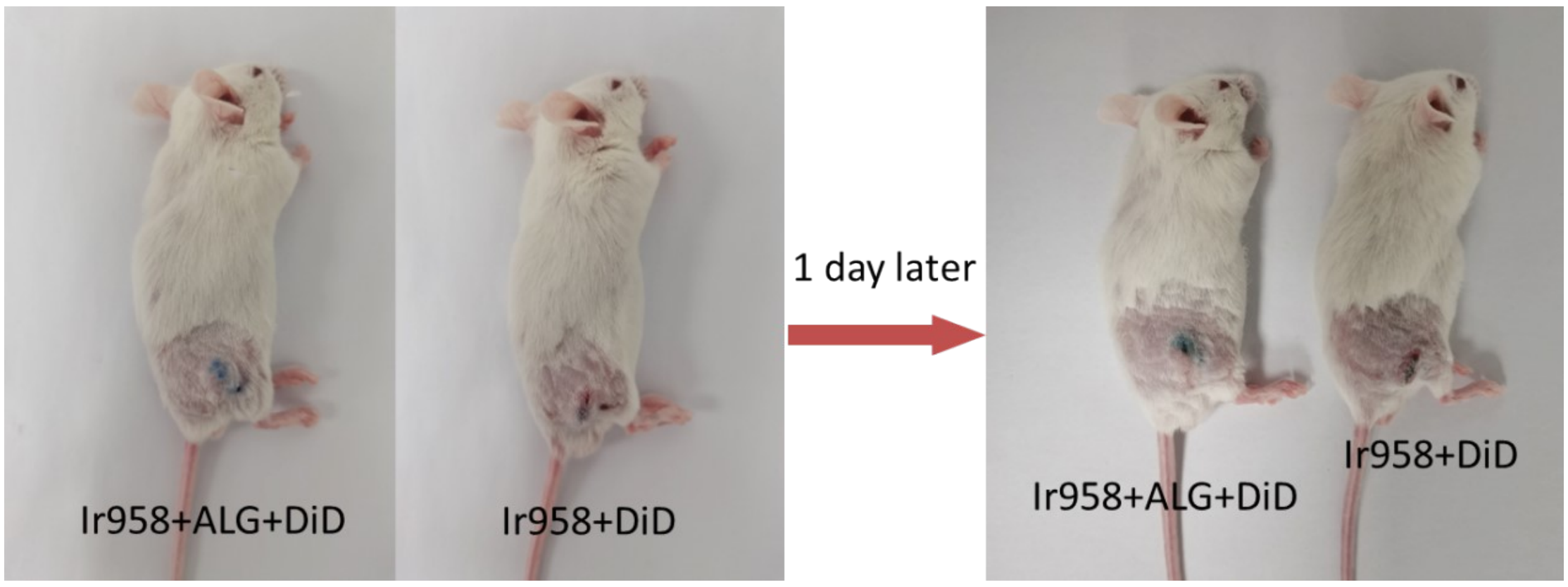

Figure S8. *In vivo* images of mice immediately after intratumoral injection of Ir958+DiD and ALG/Ir958+DiD, as well as image acquired 1-day post-injection, demonstrating the localization and immobilization effect of the hydrogel at the tumor site.

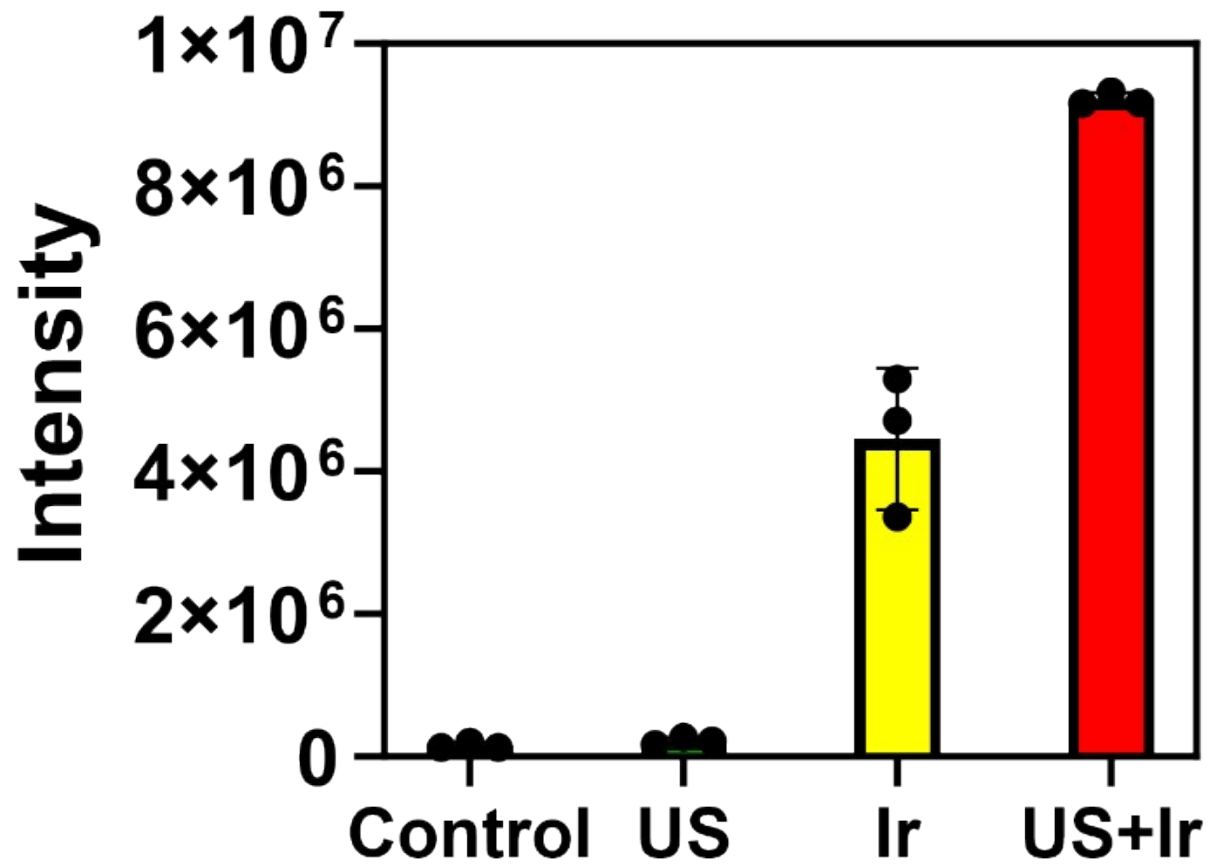

Figure S9. Comparison of intratumoral HIF-1α fluorescence intensity in BALB/c mice of different groups.

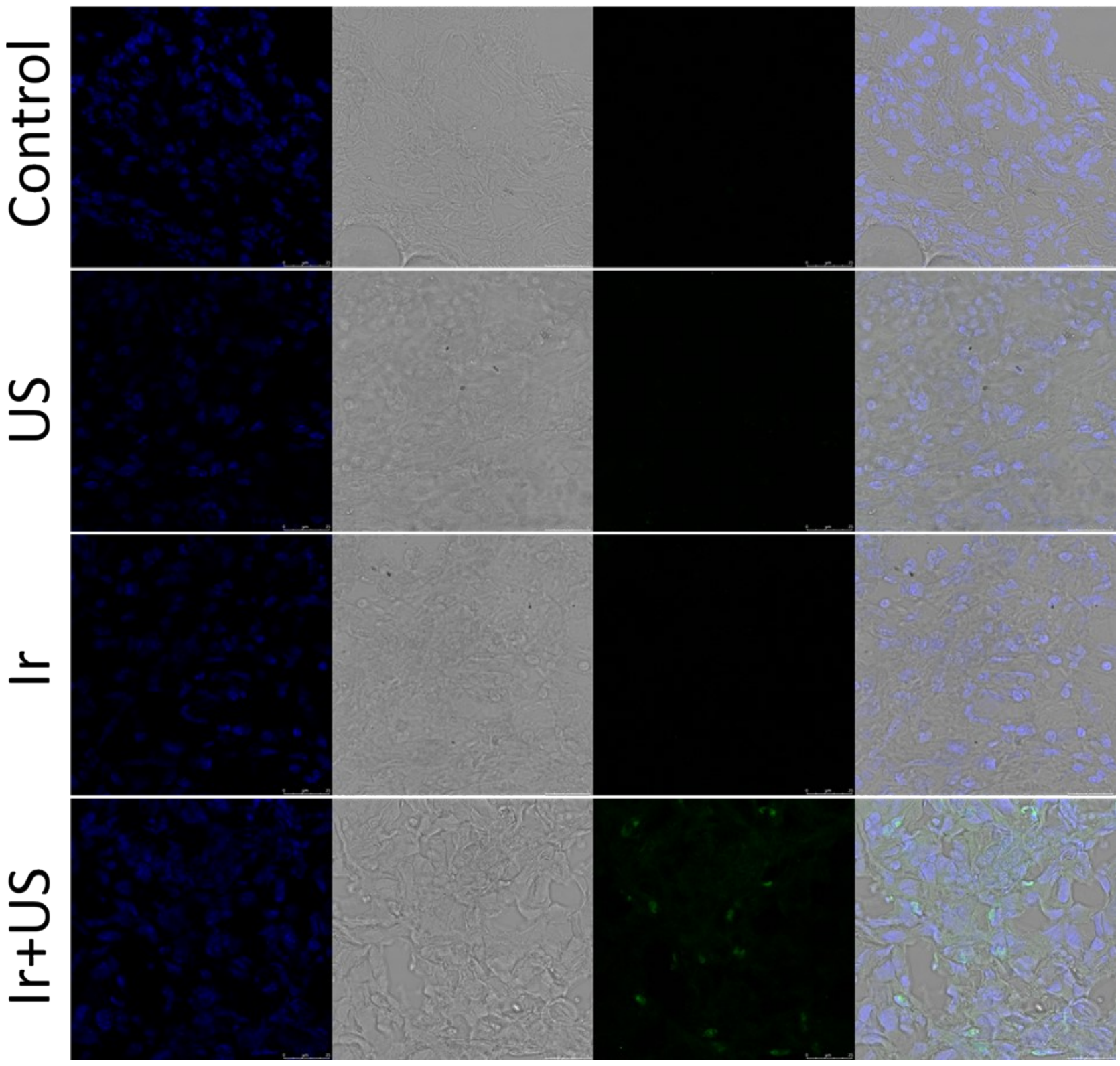

Figure S10. CLSM images of DCFH-DA-stained tumor tissues harvested from mice receiving distinct treatments. Blue fluorescence represents DAPI-labeled cell nuclei.

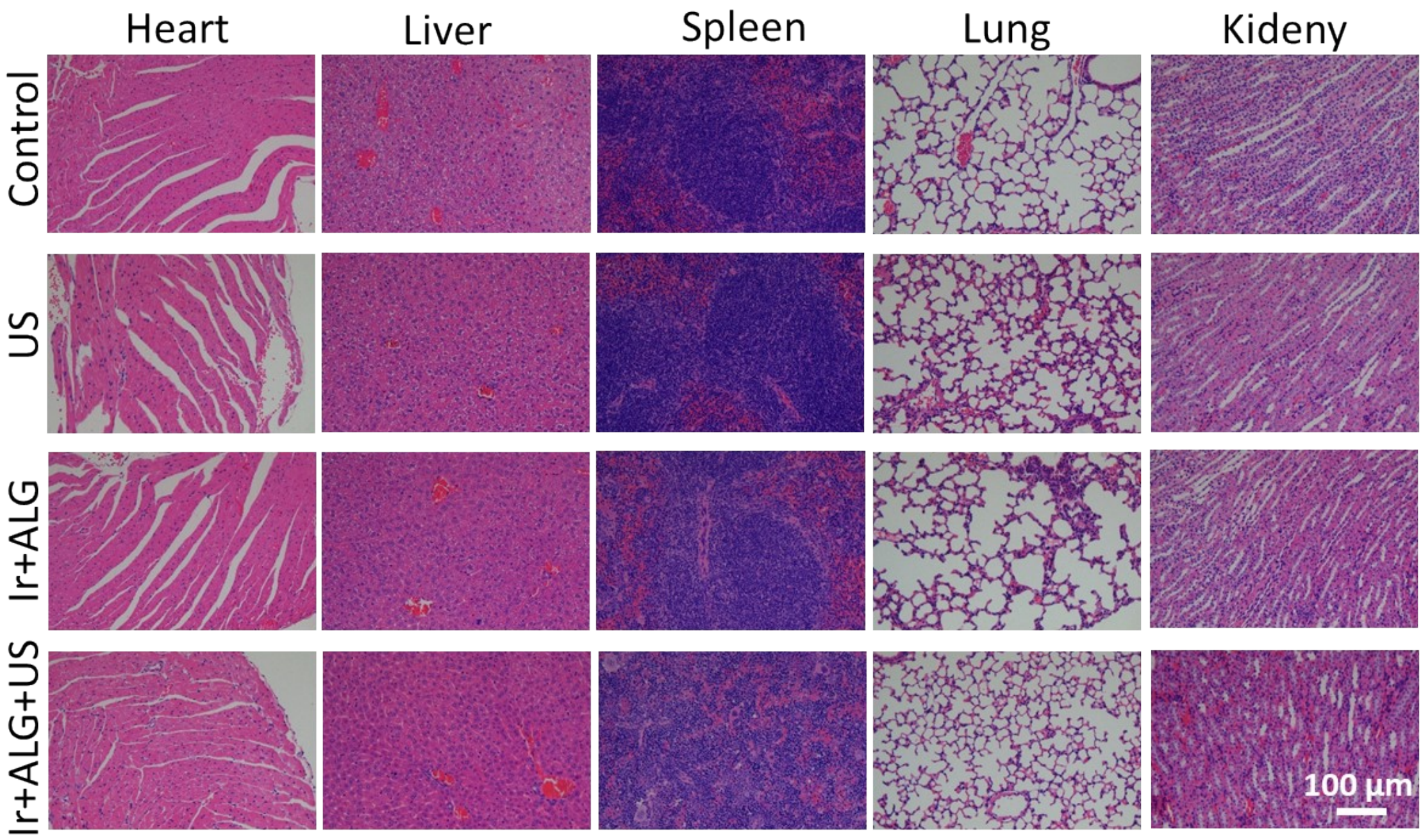

Figure S11. H&E staining images of major organs from BALB/c mice in different groups at 24 h post-treatment.
